# Vaginal Fistulas of the Bladder and Small Bowel After Two-Dimensional Intracavitary Brachytherapy in a Patient With Cervical Cancer

**DOI:** 10.7759/cureus.11537

**Published:** 2020-11-17

**Authors:** Yuki Yamada, Natsuo Tomita, Yuto Kitagawa, Mikiko Imai, Mitsuaki Ito

**Affiliations:** 1 Radiation Oncology, Kasugai Municipal Hospital, Kasugai, JPN; 2 Radiology, Nagoya City University Graduate School of Medical Sciences, Nagoya, JPN; 3 Radiology, Japanese Red Cross Nagoya Daini Hospital, Nagoya, JPN; 4 Obstetrics and Gynecology, Kasugai Municipal Hospital, Kasugai, JPN

**Keywords:** brachytherapy, radiotherapy, toxicity, two-dimensional, uterine cervical neoplasms

## Abstract

Image-guided brachytherapy (IGBT) is commonly used for patients with cervical cancer, but two-dimensional intracavitary brachytherapy (2D-ICBT) is also still utilized for certain patients. We report a patient with cervical cancer who developed vaginal fistulas of the bladder and small bowel after chemoradiotherapy with 2D-ICBT. A 61-year-old woman with stage IIB cervical cancer underwent a combination of external beam radiotherapy (EBRT) at a dose of 50.4 Gy in 28 fractions and 2D-ICBT at a dose of 22 Gy in four fractions. As packs were well inserted around the uterus in all fractions of 2D-ICBT, the doses to the surrounding organs at risk (OAR) could be likely to be kept at low levels. She developed a huge fistula between the vagina and bladder approximately 2.5 years after radiotherapy (RT). She also developed a fistula between the vagina and small bowel approximately seven years after RT and underwent bypass from the small bowel to the transverse colon. The OAR were delineated using computed tomography for EBRT planning, and the cumulative dose of 2D-ICBT plus EBRT was evaluated as the source of toxicity. The cumulative dose converted to the equivalent dose in 2-Gy fractions (EQD2) was calculated using the linear-quadratic model with α/β = 3 for the OAR. The cumulative EQD2 values of the minimum dose to the most irradiated 2 cc (D2cc) of the bladder and small bowel were 90.2 Gy and 79.5 Gy, respectively. These values exceeded the upper limits of the dosimetric criteria of the OAR, suggesting an association with both vaginal fistulas. As the adoption of IGBT is too slow in some countries, it is noteworthy that a reduced bladder volume may result in a significant increase in the dose to the small bowel and bladder in 2D-ICBT.

## Introduction

Both external beam radiotherapy (EBRT) and intracavitary brachytherapy (ICBT) are integral components of definitive radiotherapy (RT) for patients with cervical cancer [1]. Image-guided brachytherapy (IGBT) is an ICBT technique with a three-dimensional treatment plan using computed tomography (CT) or magnetic resonance imaging (MRI). IGBT can be adjusted to the size and shape of the tumor while minimizing the dose to the organs at risk (OAR) [2]. Several studies reported improvement of oncological outcomes and the reduction of toxicities with IGBT compared with two-dimensional (2D)-ICBT [3,4]. Thus, IGBT is employed worldwide for the treatment of cervical cancer. On the other hand, 2D-ICBT is also used for certain patients in some countries. For example, the Japanese national survey of ICBT recently revealed that 40% of Japanese patients are still treated by 2D-ICBT [5], probably because of Japanese structural issues regarding radiation oncology [6]. In fact, there are only a few reports for IGBT in most Asian countries. As only 2D-ICBT is available at our institution, the dose-volume data of the OAR and the target cannot be obtained. We report a patient with cervical cancer who developed severe adverse effects, such as vaginal fistulas of the bladder and small bowel, after chemoradiotherapy and the possible causes. The present study followed the ethical standards laid down in the 1964 Declaration of Helsinki and its later amendments. This study was performed after approval by the Institutional Review Board of our hospital (Approval Number: 424) in line with the Ethical Guidelines for Medical and Health Research Involving Human Subjects in Japan.

## Case presentation

**Figure 1:**
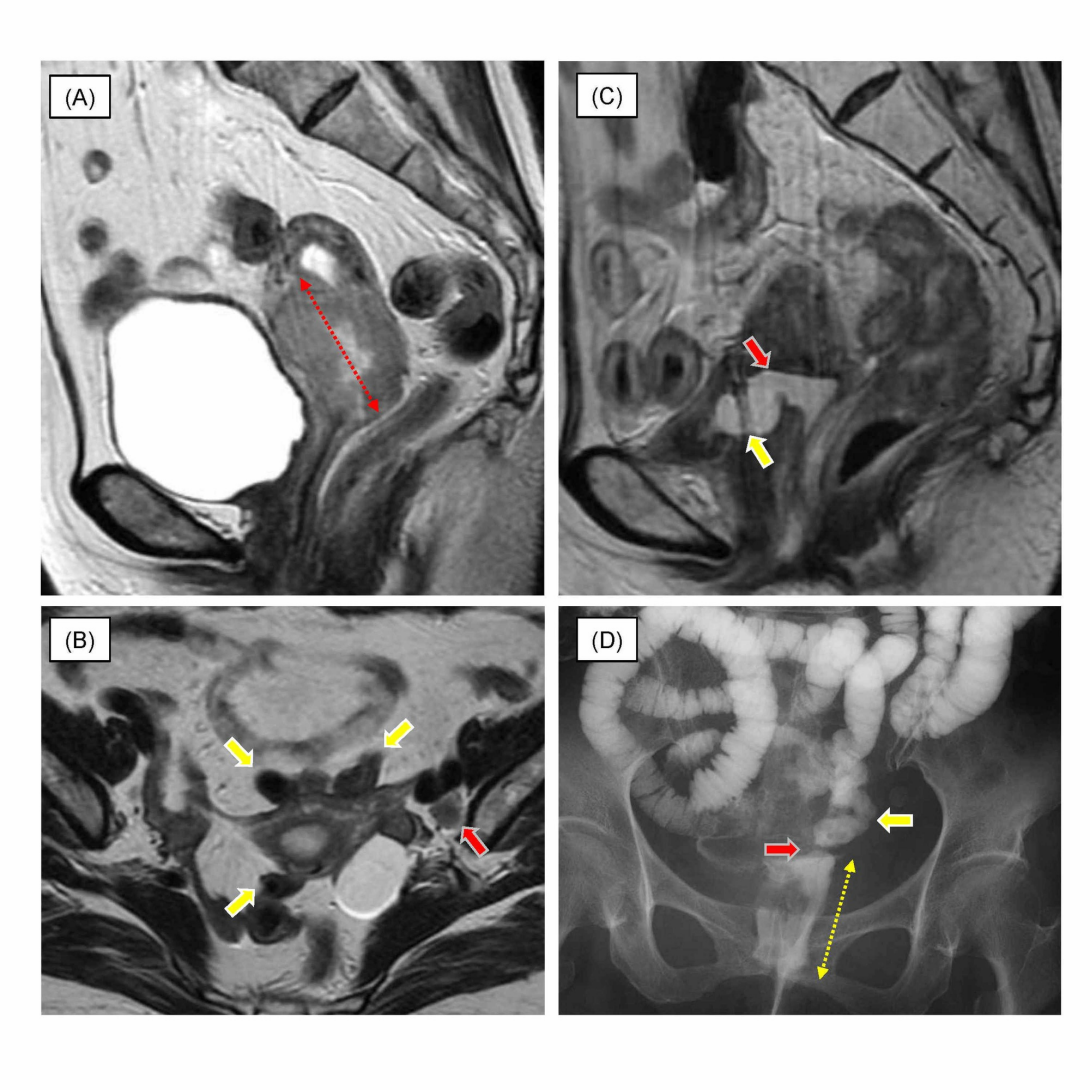
Magnetic resonance imaging (MRI) of the pelvis and radiographic enteroclysis. T2-weighted MRI of the pelvis before radiotherapy (RT) (A, B), and at two years and five months after RT (C). Radiographic enteroclysis with gastrografin at seven years and two months after RT (D). Primary tumor (A, arrow). Swelling of the left internal iliac node (B, red arrow) and the small bowel around the uterus (B, yellow arrow). Site of the fistula between the vagina and bladder (C, red arrow), and the bladder (C, yellow arrow). Site of the fistula between the vagina and small bowel (D, red arrow), and the small bowl (D, yellow arrow) and vagina (D, yellow dotted arrow).

A 61-year-old woman presented to the Department of Obstetrics and Gynecology of our hospital with post-menopausal bleeding. She underwent hysteroscopy and a cervical tumor with parametrial invasion was found. Biopsy of the cervical lesion confirmed the diagnosis of squamous cell carcinoma. Pelvic MRI detected a 51-mm cervical tumor and swelling of the left internal iliac node (Figure 1).

The patient had no evidence of metastasis to a distant organ on enhanced CT. She was clinically diagnosed with stage IIB by the International Federation of Gynecology and Obstetrics (FIGO) 2008 staging criteria for cervical cancer. She had no notable medical history.

**Figure 2:**
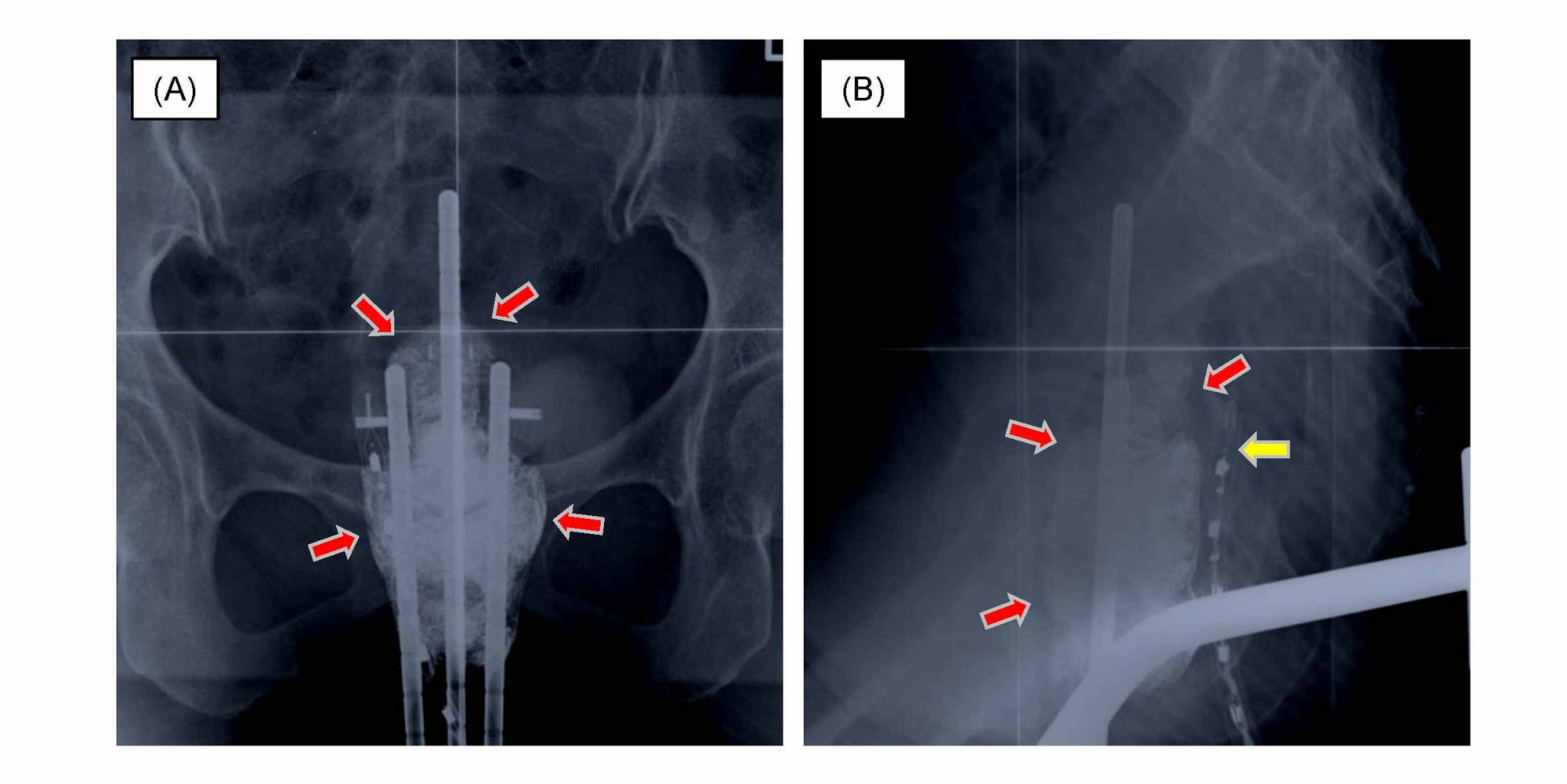
Two-way radiographs of two-dimensional intracavitary brachytherapy. Inserted packs between the uterus and rectum or bladder (A, B, red arrows), and the dosimeter to the rectum (B, yellow arrow).

The patient was treated by chemoradiotherapy, and underwent a combination of EBRT and 2D-ICBT. For EBRT, the patient was simulated by CT with a 5.0-mm slice thickness and treated using a linear accelerator with a 10-MV photon beam. An initial dose of 30.6 Gy in 17 fractions was delivered to the entire pelvis using a 4-field box technique, and 19.8 Gy in 11 fractions was subsequently delivered to the entire pelvis with a 3.0-cm-wide central shield through anterior-posterior and posterior-anterior ports. A total dose of 50.4 Gy was prescribed to the entire pelvis in 1.8-Gy fractions. 2D-ICBT was performed with a 2D plan using two-way radiographs and was applied after 34.2 Gy of EBRT. 2D-ICBT was not administered on the same day as EBRT. The dose prescribed to point A was selected according to the Manchester System. The patient was treated using standard intracavitary applicators without interstitial implants using a 60Co remote afterloading system (microSelectron; Elekta, Stockholm, Sweden). The total dose of 2D-ICBT was 22 Gy in four fractions once a week. Two-way radiographs at the first round of 2D-ICBT are shown in Figure 2.

Packs were well inserted between the uterus and rectum or bladder in all fractions. The doses of the rectum could be likely to be kept at low levels according to the ICRU-38 guideline [7]. As the catheter balloon was not inserted in 2D-ICBT at the wish of the patient, the doses of the bladder were not recorded. A 5-fluorouracil/cisplatin (FP) regimen was administered in the concurrent (two courses) and adjuvant phases (three courses) with RT once every four weeks. The FP regimen consisted of 5-fluorouracil (700 mg/m2 intravenously) on days one to four and cisplatin (70 mg/m2 intravenously) on day one. Chemoradiotherapy was performed successfully without acute major complications or prolonged RT period. The patient achieved a complete response at the end of RT. The patient was followed closely without anticancer therapy.

She presented with gross hematuria and incomplete urinary retention one year after the completion of RT. The patient received endoscopic mucosal cauterization for hemorrhagic radiation cystis. She benefited symptomatically from the cauterization but developed urinary leakage thereafter. MRI at two years and five months after RT demonstrating the fistula between the vagina and bladder is shown in Figure 1. She had recurrence at the vulva at four years and seven months after RT, and received six courses of combination therapy with carboplatin (area under the curve of 5 mg/ml/min) and paclitaxel (175 mg/m2) on day one of a 28-day cycle. She developed leakage of diarrhea stool from the vagina at seven years and two months after the completion of RT. The obstetrician-gynecologist confirmed the fistula in the inmost recesses of the left side of the vagina. The fistula between the vagina and small bowel on radiographic enteroclysis with gastrografin is shown in Figure 1. She underwent bypass from the small bowel to the transverse colon. She died due to progressive cervical cancer eight years after RT.

**Figure 3:**
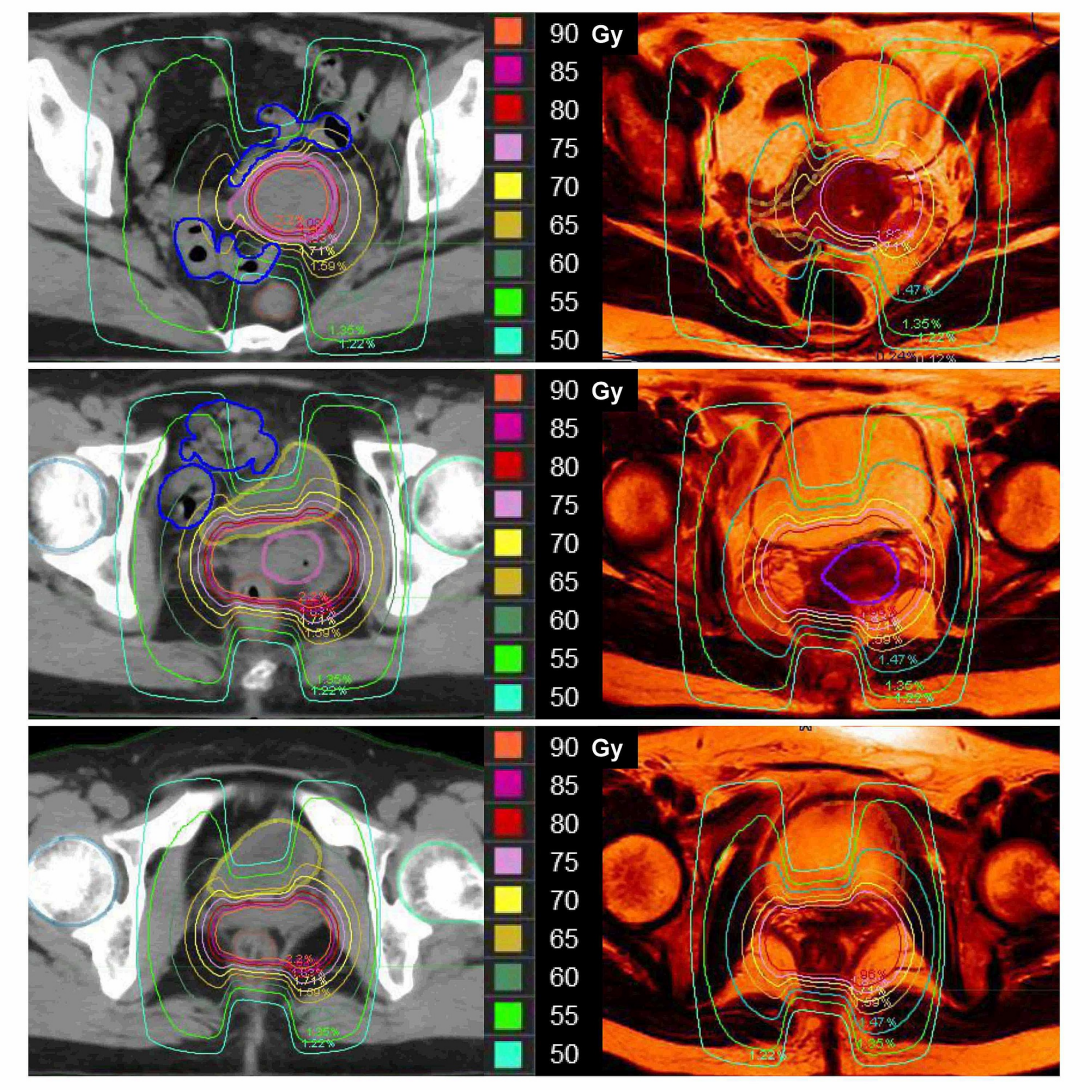
Comparison of dose distribution and dose volume histogram between the small (A) and large bladder (B) for the patient with cervical cancer.

We investigated the cause of the two severe adverse effects: vaginal fistulas of the bladder and small bowel. The OAR and high-risk clinical target volume (HR-CTV) were delineated using CT for EBRT planning according to the guidelines of the Japanese Radiation Oncology Study Group [8]. The minimum dose delivered to 90% (D90) of the HR-CTV and other dose-volume histogram (DVH) parameters were calculated hypothetically using the same dwell position. The cumulative dose of HDR-ICBT plus EBRT converted to the equivalent dose in 2-Gy fractions (EQD2) was calculated using the linear-quadratic model with α/β = 10 for the HR-CTV and α/β = 3 for the OAR. Dose parameters including D2cc to the OAR and HR-CTV D90 were calculated. The DVH and dose distributions were theoretically reproduced on the EBRT planning CT (Figure 3).

The cumulative EQD2 values of the D90% of the HR-CTV and D2cc of the OAR were as follows: 73.6 Gy for the HR-CTV, 90.2 Gy for the bladder, and 79.5 Gy for the small bowel. The cumulative EQD2 values of the D2cc of the OAR were associated with the severe adverse effects, such as vaginal fistula into the bladder or small bowel, because these values exceeded both goals and upper limits of the dosimetric criteria: < 80 Gy and < 90 Gy for the bladder, respectively; < 60 Gy and < 75 Gy for the small bowel, respectively [2]. The bladder volume was only 33.8 cc, as shown in Figure 3. The reduced bladder volume may have resulted in a significant increase in the high-dose areas of the small bowel and bladder.

## Discussion

We report a rare case of vaginal fistulas of the bladder and small bowel in a patient with cervical cancer treated by chemoradiotherapy, which consisted of EBRT and 2D-ICBT in combination with five cycles of a FP regimen. As packs were well inserted around the uterus in all fractions of 2D-ICBT, the doses to the OAR could be likely to be kept at low levels. When patients develop such severe adverse effects, they usually have advanced disease or poor response during RT and high dose must be delivered to the tumor. Our present patient received a total dose of 50.4 Gy in EBRT, but 19.8 Gy was delivered to the entire pelvis with a 3.0-cm-wide central shield. In our case, there were several potential causes of the development of vaginal fistulas. The root cause was the high doses to the OAR beyond the upper limit. The cumulative EQD2 values of the D2cc of the OAR were associated with the severe adverse effects, such as vaginal fistula into the bladder or small bowel, because these values exceeded both goals and upper limits of the dosimetric criteria: < 80 Gy and < 90 Gy for the bladder, respectively; < 60 Gy and < 75 Gy for the small bowel, respectively [2]. The dose-volume data of the OAR were unavailable during 2D-ICBT. The reduced bladder volume was considered to be the main factor for the high doses to the small bowel. The bladder volume was only 33.8 cc, as shown in Figure 3A. The comparison of dose distributions between Figure 3A and Figure 3B showed that the reduced bladder volume resulted in a significant increase in the high-dose areas of the small bowel. A few studies have reported the effects of bladder fullness on dose distribution to the bladder and small bowel [9,10]. An increase in bladder volume resulted in a significant reduction in the hot spot dose to the small bowel at the expense of an increase to the bladder without changing the dose distribution to the rectum. On the other hand, the D2cc of the bladder were almost the same between Figure 3A and Figure 3B. However, the high-dose areas of the bladder seemed to be larger in Figure 3A than Figure 3B because of the shriveled bladder in Figure 3A. An inadequate central shield may also result in high doses to the small bowel. The small bowel was in close contact with the uterus in our patient, as shown in Figure 1. As shown in Figure 3A, high doses were administered to the small bowel on the sides of the uterus; therefore, a 3.0-cm central shield may have been insufficient to protect the small bowel.

Kim et al. performed a systematic review and meta-analysis of the impact of ICBT technique (2D or IGBT) on outcomes of cervical cancer patients [11]. The pooled hazard ratio (HR) regarding toxicity was evaluated in five cohorts in three studies, and the HR of IGBT compared to 2D-ICBT was 0.54 (95% confidence interval 0.37-0.77). Lin et al. reported long-term outcomes of patients with cervical cancer who were treated with intensity modulated radiation therapy and IGBT (IMRT/IGBT) compared with those treated with conventional EBRT and 2D-ICBT (2D EBRT/ICBT) [12]. The ratio of grade > 3 late bowel or bladder toxicities in the 2D EBRT/ICBT (n = 600) vs IMRT/IGBT (n = 300) groups was 18.3% vs 14.7% (P = 0.01). Although vaginal fistulas of the bladder or rectum were found in a few patients, no vaginal fistula of the small bowel was observed after IGBT [12,13]. A vaginal fistula of the small bowel was previously unreported even after 2D-ICBT, too [14,15]. Thus, a vaginal fistula of the small bowel seems to be fairly uncommon in cervical cancer patients after RT.

Intensive chemotherapy and the endoscopic mucosal cauterization for hemorrhagic radiation cystis may also be associated with the development of severe adverse effects. In our country, patients usually receive five courses of single-agent weekly cisplatin (40 mg/m2) concurrently [16]. The severe complication is uncommon in the endoscopic mucosal cauterization such as Argon plasma coagulation, and the incidence of perforation was reported to be about 3% [17]. Although the endoscopic mucosal cauterization could be associated with a fistula between the vagina and bladder, we think that the fistula happened due to the overdose to the bladder because the cystoscope showed the ulcer in the bladder. Late radiation cystitis can develop from six months to 20 years after RT [18]. ‘Only one year after treatment’ is earlier than the common onset timing. In addition, previous studies have shown a correlation between high-dose irradiation to the bladder and the incidence of radiation-induced hemorrhagic cystitis [2]. These facts make the hypothesis that the bladder was exposed to high-dose irradiation more certain. The theoretical reproduction of the ICBT dose distribution is a limitation in our case.

## Conclusions

We reported a patient with cervical cancer who developed vaginal fistulas of the bladder and small bowel after chemoradiotherapy with 2D-ICBT. The doses to the OAR were unable to be obtained for 2D-ICBT. The hypothetical cumulative doses to the bladder and small bowel exceeded the upper limits of the dosimetric criteria, suggesting an association with the vaginal fistulas. The reduced bladder volume may have resulted in a significant increase in the dose to the small bowel and bladder. As the adoption of IGBT is too slow in some countries, physicians should be aware of the effects of bladder fullness on dose distribution to the bladder and small bowel because of indeterminable doses to the OAR.
